# Fear of unintended pregnancy and sexual quality of life during the menopausal transition in a Turkish population: A cross-sectional study of associated factors

**DOI:** 10.1371/journal.pone.0353656

**Published:** 2026-07-14

**Authors:** Aysun Badem

**Affiliations:** Department of Nursing, Department of Women’s Health Nursing, Faculty of Health Sciences, Kahramanmaraş Sütçü İmam University, Kahramanmaraş, Türkiye; Tarbiat Modares University Faculty of Medical Sciences, IRAN, ISLAMIC REPUBLIC OF

## Abstract

**Objective:**

This study aimed to investigate fear of unintended pregnancy, contraceptive method use, sexual quality of life, and associated factors among perimenopausal women.

**Methods:**

This descriptive cross-sectional study was conducted in 2025 with 124 perimenopausal women aged 40–50 years. The data have been collected using a sociodemographic-obstetric questionnaire and the Turkish version of the Sexual Quality of Life-Female (SQOL-F) scale. Mann–Whitney U, Kruskal–Wallis H and Linear regression analysis tests were applied.

**Results:**

The median SQOL-F score was 84.50. The score indicated a moderate-to-high level of sexual quality of life. Significant differences in SQOL-F scores were observed according to employment status, education level, parity, mode of delivery, childbirth experience, and fear of unintended pregnancy (p < 0.05). Relatively higher SQOL-F scores have been exposed by women with higher education levels, lower parity, cesarean delivery, and positive birth experiences. Significantly lower SQOL-F scores have been observed on the fear of unintended pregnancy predicated women.

**Conclusions:**

The fear of unintended pregnancy is prevalent among perimenopausal women and is associated with lower sexual quality of life. Comprehensive nursing interventions may be critical including contraceptive counseling and sexual health education tailored to the needs of perimenopausal women.

## Introduction

Perimenopause is defined as a transitional stage in a woman’s reproductive lifespan between the ages of 40 and 50 before the menopause [1]. The Perimenopause is characterized by hormonal fluctuations and irregular menstrual cycles. Physical, psychological, and sexual symptoms often accompany in this period [1]. Although many women at this stage have completed their reproductive plans, the biological possibility of pregnancy remains. Therefore, the continuing risk of unintended pregnancy may lead to anxiety or fear in some women.

Sexuality is a fundamental component of health and is strongly associated with quality of life [2]. Women in the perimenopausal period are especially vulnerable to depressive symptoms and lower life satisfaction [3]. Sexual satisfaction is influenced by factors beyond physiological functioning [2]. Contraception is one of these factors and may either enhance or negatively affect sexual experiences depending on individual perceptions and side effects [4]. Therefore, sexual quality of life is shaped by the interaction of psychosocial and physiological factors [5].

Pregnancy risk remains a critical yet often overlooked dimension of sexual health during the perimenopausal period. Pregnancies occurring at the end of the reproductive period are associated with increased risks of complications such as preeclampsia, gestational hypertension, gestational diabetes, and fetal anomalies [6]. In addition, the higher likelihood of miscarriage may further intensify women’s concerns about conception and its outcomes [7]. In addition to physical risks, the possibility of unintended pregnancy may also have psychological consequences. Women in this life stage often experience multiple stressors, including professional responsibilities, family roles, age-related changes, and the transition toward menopause [3]. Despite declining fertility, many women remain sexually active in their late 40s and 50s, yet contraceptive counseling during this period is frequently insufficient [8]. This gap may increase the risk of unintended pregnancy and associated anxiety. The continued risk of pregnancy may impose a substantial emotional burden and negatively affect overall well-being and sexual life [8].

The fear of unintended pregnancy (FUIP) in perimenopausal women can be explained through cognitive-behavioral theory. Negative thoughts about potential future events such as “What if I get pregnant again?” can generate negative emotional responses and arise the anxiety. The cognitive patterns may lead to sexual avoidance, dissatisfaction, and a diminished sense of sexual safe during the perimenopausal period [9]. Therefore, FUIP can be recognized as both a cognitive and emotional phenomenon, rather than solely a physiological concern. Hence, the combined the worries about fertility and the FUIP may contribute to massively increase the anxiety levels [10]. Also, declining estrogen levels during perimenopause are related with a range of sexual dysfunctions, including reduced libido [4], vaginal dryness and dyspareunia [11], difficulties with arousal and orgasm [12], psychological distress [8], and broken communication with partners.

During the menopausal transition some women may experience increased anxiety about contraceptive effectiveness, while others may underestimate their risk of pregnancy and reduce contraceptive use. In addition, the FUIP may also be formed by previous reproductive experiences, the quality of the partner relationship, and personal perceptions of health. Although the majority of existing research has concentrated on the menopausal period, some studies have investigated the effect of physiological changes [13] and transitional symptoms specific to perimenopause on women’s sexual health [14]. However, emotional factors especially FUIP has not yet been examined in this context. In addition, there exist limited studies focused on contraceptive decision-making and perceptions of unintended pregnancy risk among women in this age group [15].

The FUIP may affect sexual well-being during perimenopause, making it important to examine this relationship. The FUIP has received limited examination by researchers as an emotional factor. The Reproductive Life Planning (RLP) define the importance of addressing both fertility intentions and contraceptive needs throughout a woman’s reproductive life course [16]. In this situation, evaluating FUIP together with contraceptive preferences is critical for exposing the reproductive health needs of women in perimenopause. In this study, fear of unintended pregnancy, contraceptive use, and sexual quality of life in perimenopausal women are examined.

## Materials and Methods

### Study design

This cross-sectional descriptive study was conducted between June and July 2025 with 124 perimenopausal women. Participants who met the inclusion criteria completed an online survey via Google Forms. The required sample size was calculated using G*Power (version 3.1), a statistical software for power analysis, based on two methodologically similar studies [15,17], assuming an effect size of 0.3, a significance level of 0.05, and a statistical power of 95%, which indicated a minimum of 122 participants. Participants were recruited through convenience (volunteer) and snowball sampling methods. Data were collected using an online Google Forms survey distributed via social media and messaging platforms. All questions were set as mandatory; therefore, no missing data were observed, and incomplete responses were not recorded by the survey system. A total of 138 women initially responded to the survey; however, 14 were excluded after eligibility screening for not meeting the inclusion criteria, such as age range and marital status.

### Inclusion Criteria

In this study, women aged 40–50 years who were married, literate, sexually active (i.e., currently engaging in sexual intercourse), and in the perimenopausal phase (i.e., had experienced menstruation within the past 12 months) have been include. Participants were included if they voluntarily agreed to participate and met the inclusion criteria.

### Exclusion Criteria

Women were excluded if they had physical disabilities that could affect sexual functioning, diagnosed psychiatric disorders, a history of gynecologic or breast cancer, recent vaginal infections, or prior pelvic surgery. These criteria were established to minimize potential confounders that could directly impact sexual functioning or psychological well-being.

### Descriptive and obstetric characteristics of the women

Data on sociodemographic characteristics included age, employment status, education level, income status. Reproductive characteristics included the number of pregnancies, number of living children, miscarriage and curettage history, experience with unintended pregnancy, mode of delivery, childbirth complications, fertility intentions, FUIP, anticipated emotional reaction to a potential pregnancy, reasons for avoiding pregnancy, current use of contraceptive methods, and type of contraceptive method used. Fear of unintended pregnancy was assessed using a single self-report item: “Are you afraid of becoming pregnant unintentionally?” with response options “Yes” or “No.” These variables were considered independent variables in the analysis.

### Questionnaire Scales- Sexual Quality of Life-Female (SQOL-F) Scale

The Sexual Quality of Life-Female (SQOL-F) Scale, originally developed by Symonds et al. (2005) [13], was adapted into Turkish by Tuğut and Gölbaşı in 2010 [14]. It consists of 18 items rated on a six-point Likert scale (1 = strongly agree to 6 = strongly disagree). Total scores range from 18 to 108, with higher scores indicating better sexual quality of life. Items 1, 5, 9, 13, and 18 are reverse scored. No cut-off point is defined for the scale. In the present study, the Cronbach’s alpha coefficient was 0.928, indicating excellent internal consistency.

### Statistical Analysis

Statistical analyses were conducted using IBM SPSS Statistics for Windows, Version 20.0 (IBM Corp., Armonk, NY, USA). Continuous variables were summarized as median, interquartile range (IQR: Q1–Q3), minimum, and maximum; categorical variables were presented as frequencies and percentages. The Kolmogorov–Smirnov and Shapiro–Wilk tests indicated a non-normal distribution of SQOL-F scores (p < 0.05). Therefore, non-parametric tests were used: Mann–Whitney U test for comparisons between two groups, and Kruskal–Wallis H test for more than two groups. Post hoc pairwise comparisons were performed using Bonferroni-adjusted Mann–Whitney U tests where applicable. Effect sizes were calculated and reported as r and η². Statistical significance was set at p < 0.05. Univariable and multivariable linear regression analyses were conducted to evaluate factors associated with SQOL-F scores. Variables were selected a priori based on clinical relevance and previous literature and were entered simultaneously into the multivariable linear regression model. Regression assumptions were evaluated using diagnostic plots (histogram and normal P–P plot) of standardized residuals. Multicollinearity was assessed using variance inflation factors (VIF), and all VIF values were < 2.

## Results

### Sociodemographic and Obstetric Characteristics of the Participants and Total SQOL-F Scores

The normality of the data distribution was assessed using the Kolmogorov–Smirnov (p = 0.018) and Shapiro–Wilk (p = 0.001) tests, both of which indicated a non-normal distribution. Consequently, all group comparisons were performed using non-parametric tests, specifically the Mann–Whitney U and Kruskal–Wallis H tests. Detailed information on the participants’ sociodemographic, educational, and obstetric characteristics is presented in Table 1.

**Table 1:** Sociodemographic, obstetric and reproductive characteristics of the participants (n = 124).

Variable		n	%
**Age**	40–45	60	48.4
	46–50	64	51.6
**Employment status**	Employed	51	41.1
	Unemployed	73	58.9
**Education**	Primary/Secondary	50	40.3
	High school	23	18.5
	University or higher	51	41.1
**Income status**	Low	39	31.5
	Medium	69	55.6
	High	16	12.9
**Gravida**	0–3	73	58.9
	4 or more	51	41.1
**Living children**	0–3	101	81.5
	4 or more	23	18.5
**Satisfaction with number of children**	As desired	93	75.0
	Less than desired	23	18.5
	More than desired	8	6.5
**Miscarriage history**	Yes	48	38.7
	No	76	61.3
**Curettage history**	Yes	35	28.2
	No	89	71.8
**Unintended pregnancy**	Yes	39	31.5
	No	85	68.5
**Mode of delivery**	Vaginal	61	49.2
	Cesarean	63	50.8
**Birth experience**	Difficult	25	20.2
	Moderate	88	71.0
	Easy	11	8.8
**Fear of unintended pregnancy**	Yes	66	53.2
	No	58	46.8
**Pregnancy intention**	Yes	9	7.3
	No	115	92.7
**Emotional response if found to be pregnant**	Happiness	18	14.5
	Sadness	52	41.9
	Uncertainty	54	43.5
**Planned action if pregnancy occurs**	Don’t know	46	37.1
	Give birth	42	33.9
	Get depressed	21	16.9
	Have curettage	7	5.6
	Other	8	6.5
**Suspicion of pregnancy during menstrual delay**	Yes	49	39.5
	No	75	60.5
**Reasons for avoiding pregnancy**	Unable to care	22	17.7
	Don’t want	18	14.5
	Advanced age	31	25.0
	Health risk	4	3.2
	Financial issue	4	3.2
	Already grandmother	2	1.6
**Using contraceptive**	Yes	71	57.3
	No	53	42.7
**Mode of contraceptive**	Condom	30	24.2
	Intrauterine Device (IUD)	17	13.7
	Tubal ligation	23	18.5
	Hormone pill/injection	3	2.4
	Withdrawal	26	21.0
	Other	25	20.2

The age of the 124 participants ranged from 40 to 50 years. The majority (55.6%) reported a medium income level. Most participants (75%) were satisfied with the number of children they had. A history of miscarriage was reported by 38.7%, and curettage history by 28.2%, while 31.5% reported unintended pregnancies. In terms of subjective birth experience, 20.2% described it as difficult (Table 1).

A total of 92.7% of participants reported no desire to become pregnant again, and 53.2% expressed FUIP. When asked about their emotional reaction to a potential pregnancy, 41.9% stated they would feel sadness. Most participants (83.1%) perceived pregnancy at this stage as risky. The most commonly cited reasons for avoiding pregnancy were advanced maternal age (25.0%) and inability to care for a child (17.7%). Contraceptive use was reported by 57.3% of women, with condoms (24.2%), withdrawal (21.0%), and tubal ligation (18.5%) being the most frequently used methods (Table 1). The median SQOL-F score was 84.5 (range: 37–108; IQR: 67.0–97.0) (Table 2).

**Table 2:** Total score of the Sexual Quality of Life-Female Scale (SQOL-F).

	n	Median	IQR	Min-Max
**SQOL-F Total score**	124	84.50	67.00–97.00	37.00–108.00

### Differences in SQOL-F Scores by Independent Variables

SQOL-F scores did not significantly differ by age, number of living children, satisfaction with number of children, history of miscarriage or curettage, unintended pregnancy, pregnancy intention, perceived pregnancy risk, emotional response to a potential pregnancy, suspicion of pregnancy during menstrual delay, or contraceptive use (p > 0.05). However, significant differences were identified based on employment status and educational level (p < 0.05), with lower scores particularly among those with primary or secondary education. Parity was also associated with SQOL-F scores, with women who had 0–3 pregnancies reporting higher scores than those with four or more (p < 0.05). Additionally, mode of delivery and perceived birth experience were significantly related to SQOL-F scores (p < 0.05). Participants who reported FUIP had significantly lower sexual quality of life scores compared to those without such fear (p < 0.05) (Table 3).

**Table 3:** Analysis of Total SQOL-F Scores by Independent Variables.

Variables		SQOL-F Median [IQR])	Mean Rank	Test	Effect size r/ η²	p
**Age**	40–45	85.00		U = 1865.50	r = .02	0.785
	46–50	84.50		z = –0.27		
**Employment status**	Employed	87.00	70.57	U = 1450.00	r = .19	**0.037**
	Unemployed	80.00	56.86	z = –2.09		
**Education**	Primary/Secondary (a)*	76.50	49.48	χ²=11,06 df = 2	–	**0.004**
	High school (b)	90.00	72.65			
	University or higher (c)	88.00	70.69			
	a-b *			U = 372,500 z = −2,407	r = .22	**0.016**
	a-c *			U = 826,500 z = −3,048	r = .27	**0.002**
	b-c *			U = 555,500 z = −0,362	r = .03	0.717
**Gravida**	0-3 (low to moderate parity) 4 or more (high parity)	91.00 81.00	72.36 58.30	U = 1244,500 z = −1,994	r = .18	**0.046**
**Living children**	0-3	85.00	63.19	U = 1092,000	r = .04	0.655
	4 or more	80.00	59.48	z = −0,447		
**Child satisfaction**	As desired	87.00	35.21	χ²= 2,13 df = 2	–	0.345
	Less than desired	98.00	43.20			
	More than desired	95.00	43.50			
**Miscarriage**	Yes	81.00	60.80	U = 1742,500	r = .04	0.676
	No	87.00	63.57	z = −0,418		
**Curettage**	Yes	80.00	58.63	U = 1422,000	r = .07	0.452
	No	87.00	64.02	z = −0,753		
**Unintended pregnancy**	Yes	80.00	56.42	U = 1420,500	r = .11	0.202
	No	87.00	65.29	z = −1,276		
**Mode of delivery**	Vaginal	80.00	55.80	U = 1512,500	r = .18	**0.041**
	Cesarean	87.00	68.99	z = −2,045		
**Birth experience**	Difficult	74.00	53.14	χ²=7.08 df = 2	–	**0.029**
	Moderate	84.00	62.02			
	Easy *	99.00	87.59			
	Difficult/Easy *			U = 66,500 z = −2,440	r = .42	**0.015**
	Moderate/Easy *			U = 279,000 z = −2,284	r = .20	**0.022**
**Pregnancy intention**	Yes	87.00	67.11	U = 476,000	r = .04	0.689
	No	84.00	62.14	z = −0,400		
**Fear of unintended pregnancy**	Yes	75.00	51.42	U = 1183,000	r = .33	**p < 0.001**
	No	90.50	75.10	z = −3,663		
**Emotional response if found to be pregnant**	Happiness	90.00	74.97	χ²=3,034 df = 2	–	0.219
	Sadness	83.00	62.88			
	Uncertainty	84.50	57.97			
**Suspicion of pregnancy during Menstrual delay**	Yes	78.00	57.34	U = 1584,500 z = −1,294	r = .12	0.196
	No		65.87			
**Contraceptive**	Yes	87.00	66.04	U = 1630,500 z = −1,269	r = .11	0.205
	No	82.00	57.76			

Mann–Whitney U testi:U Kruskal–Wallis H: χ² * Bonferroni post-hoc test

Multivariable linear regression model, fear of unintended pregnancy was independently associated with lower SQOL-F total scores (B = −12.66, 95% CI −18.65 to −6.84; p < 0.001). Higher education level was associated with higher SQOL-F scores (B = 4.15, 95% CI 0.67 to 8.10; p = 0.04). Current contraceptive use was associated with slightly lower SQOL-F scores (B = −1.80, 95% CI −3.38 to −0.13; p = 0.03). Age, employment status, gravida, mode of delivery, and history of unintended pregnancy were not significantly associated with SQOL-F scores (p > 0.05) (Table 4).

**Table 4:** Multivariable linear regression analysis of factors associated with SQOL-F total score.

Dependent variable	Independent variable	Coefficient (main effect)	95% CI (main effect)	p-value (main effect)
SQOL-F Total Score	Age	0.49	(−0.36, 1.28)	0.24
	Education	4.15	(0.67, 8.10)	0.04
	Employment status	1.00	(−6.19, 7.93)	0.79
	Gravida	−0.71	(−2.95, 1.78)	0.60
	Mode of delivery	0.66	(−5.87, 6.64)	0.84
	Unintended pregnancy	1.06	(−2.50, 4.51)	0.53
	Fear of unintended pregnancy (FUIP)	−12.66	(−18.65, −6.84	<0,001
	Using contraceptive	−1.80	(−3.38, −0.13)	0.03

* Adjusted unstandardized regression coefficients (B) with 95% confidence intervals are presented from a multivariable linear regression model. A two-sided p-value <0.05 was considered statistically significant. Categorical variables were entered using dummy coding with appropriate reference categories.

## Discussion

Understanding the perimenopausal period is essential to supporting women’s transition into menopause. The cultural, social, and economic factors play a major role in formed women’s perceptions to this stage beyond the intensity of menopausal symptoms, was emphasized in recently published systematic review [18]. The obtained findings indicated to sexual quality of life during perimenopause is affected by a complex interplay of sociodemographic, reproductive, and psychological factors. The obtained results on mentioned variables are discussed with the literatures as follows:

Employed women produced significantly higher SQOL-F scores compared to their unemployed women (p < 0.05) in this study. Although the most existing research has focused on postmenopausal populations, our findings may provide relevant insights into the perimenopausal period. For example, Pérez-Herrezuelo et al. (2020) found that employment was positively correlated with better sexual functioning in menopausal women [19]. Nazarpour et al. (2021) reported that employed women scored significantly higher in sexual function than housewives and retirees especially for body image and sexual health [20]. Therefore, it clearly seen that employment may contribute to improved sexual quality of life through enhanced social connectedness, financial independence, and self-efficacy.

The significantly lower SQOL-F scores have been obtained by women with primary or secondary education than women with high school or university-level education (p < 0.05). Thus, obtained result show that higher educational attainment may contribute to better sexual quality of life. The education was positively linked to sexual health outcomes have been observed in studies on postmenopausal women as similar our finding [20,21]. Although the current research focuses on an earlier reproductive stage, similar results were found in this study. High level-Educated women are more likely to have access to accurate information about fertility transitions and contraceptive methods. Therefore, this reality may enable them to make more informed health decisions and foster a stronger sense of sexual well-being.

Women with lower parity (0–3 pregnancies) demonstrated significantly higher SQOL-F scores than those with higher parity (≥4 pregnancies) (p < 0.05). The high parity (≥3–4 births) is associated with declines in sexual functions such as libido, orgasm, and overall satisfaction in the previously study. The physical consequences of repeated childbirth, including pelvic floor weakening, vaginal laxity, urinary incontinence, and prolonged postpartum recovery, may be causes of the sexual dysfunctions mentioned [22].

Grand multiparity (≥5 births) has also been related to lower scores in both sexual function and quality of life. As the same time, grand multiparity may be caused to dystocia and obesity cited as additional risk factors [23]. In comparing pregnant and non-pregnant women study, increasing parity and pregnancy status were both significantly associated with reduced sexual functioning. This finding has been suggested the pregnancy itself may be a risk factor for sexual dysfunction [24]. Hence, these findings highlight the cumulative effects of physical strain, physiological stress, and psychological burden tied to reproductive history may negatively influence women’s sexual well-being.

A significantly higher SQOL-F scores has been obtained by delivered women via cesarean section than vaginal births (p < 0.05). The obtained this result overlaps with the findings of a few recently published studies. Baud et al. (2020) observed that women who underwent elective cesarean sections experienced fewer symptoms of sexual dysfunction compared to the vaginal delivered even up to six years postpartum [25]. The reported the difference was primarily attributed to reduced pelvic floor trauma associated with cesarean birth. Furthermore, Terece et al. (2024) has found that cesarean delivery was related to higher sexual function scores and better sexual quality of life within the first year after childbirth [26].

The most participants in the sample had 0–3 births (low to moderate parity), and the distribution between cesarean and vaginal deliveries was relatively balanced. It is clearly said that the findings are based on a representative and well-distributed sample. The overall mean SQOL-F score was 84.50. Obtained score has been indicated a moderate-to-high level of sexual quality of life. Thus, this result may partly reflect the potentially protective effects of lower parity and cesarean delivery (Table 2). In this study, women with low to moderate parity and those who had cesarean deliveries reported significantly higher sexual quality of life scores. When this result is compared with existing literature indicating that higher parity may negatively affect sexual health due to increased physical strain and pelvic floor dysfunction, the obtained results is consistent [25,26]. In addition, the cesarean delivery may help preserve sexual function by minimizing pelvic trauma may be stated. Consequently, the predominance of low-to-moderate parity in this sample may have contributed to the overall favorable sexual health outcomes observed.

In this study, women who described their birth experience as “easy” reported significantly higher SQOL-F scores than those who characterized it as moderate or very difficult (p < 0.05). The results of the existing studies on analyzed the impact of childbirth experience on later sexual health are supported to this result. A systematic review by Fanshawe et al. (2023) show that traumatic delivery methods such as episiotomy and assisted vaginal birth are related to negative medium- and long-term effects on sexual function [27]. Thanks to less invasive and uncomplicated births may help preserve pelvic floor integrity, better sexual functioning and satisfaction may be provided. Moreover, subjective perceptions of childbirth have been shown to be important predictors: negatively perceived births may reduce sexual quality of life, whereas positively remembered experiences may enhance sexual quality of life [28].

The most participants have reported using at least one form of contraception (Table 1). The relatively high SQOL-F scores observed in the sample may reflect a sense of safe and sexual autonomy associated with contraceptive uses. The most commonly reported methods were condoms, tubal ligation, and withdrawal. Geleta et al. (2021) examined contraceptive use among perimenopausal women and found a usage rate of 17%, with higher rates related to older age, higher educational, and higher socioeconomic status [29]. These finding is supported to our results.

A large proportion of perimenopausal participants reported not wanting to become pregnant (92.7%), perceiving pregnancy as risky due to their age (83.1%), and anticipating feelings of sadness (41.9%) or uncertainty (43.5%) if pregnancy were to occur. This may have limited the comprehensive assessment of all dimensions of FUIP. Especially, the fear reported women had significantly lower SQOL-F scores (p < 0.05). Previous research has shown that fears related to sexual activity can adversely affect sexual function and overall quality of life. For example, Calpbinici (2024) found that a traumatic childbirth perception was related to a raised desire to avoid pregnancy and lower sexual quality of life [28]. Similarly, Phan et al. (2021) reported that pregnant women often refrained from sexual intercourse due to fears of harming the fetus. Thus, this situation leading to diminished sexual well-being [30]. While these studies involve different reproductive stages, they support the broader notion that fertility-related fears can negatively influence sexuality. Remarkably, no previous studies have directly explored the association between FUIP and sexual quality of life in perimenopausal women according to the best knowledge. Therefore, stated the novelty and contribution of the present study.

A fear of unintended pregnancy states participants during the perimenopause period exhibited significantly lower SQOL-F scores. This relationship may stem not only from fertility-related concerns but also from underlying psychological factors. A systematic review and meta-analysis by Saha et al. (2021) stated the frequent co-occurrence of mood and anxiety disorders [31]. As the same time, another review of 19 studies found a strong link between female sexual dysfunction and mood disturbances [32]. For women experiencing pregnancy-related fear, heightened anxiety combined with mood fluctuations may undermine sexual satisfaction and function. These findings emphasize that sexual quality of life is influenced not only by physiological factors but also by deeply interconnected psychological and cognitive processes.

Armeni et al. (2023) identified a high prevalence of pathological sexual dysfunction symptoms among postmenopausal women in their study on climacteric symptoms and sexual functioning [33]. A qualitative study involving women over the age of 40 revealed widespread anxiety about becoming pregnant and a strong desire to avoid conception [34]. For this age group, contraceptive needs should be addressed proactively, without requiring additional diagnostic testing to confirm perimenopausal status [35]. In the multivariable model, FUIP remained independently associated with lower SQOL-F scores after adjusting for sociodemographic and reproductive factors. These findings suggest that the relationship cannot be explained solely by potential confounding variables (Table 4). The results of this study showed that FUIP was significantly associated with sexual quality of life. The regression model explained 10.4% of the variance in sexual quality of life (R² = 0.104). Moreover, Fear of unintended pregnancy was associated with an 11.62-point lower in SQOL-F total scores (B = −11.62, SE = 3.09, p < 0.001) (Table 5). These findings highlight the need to examine sexual quality of life and its contributing factors not only in postmenopausal populations, but also throughout earlier reproductive transitions such as perimenopause.

**Table 5:** Simple linear regression analysis of the association between fear of unintended pregnancy (FUIP) and SQOL-F score.

		Regression coefficients					Model			
Dependent variable	Independent variable	**B**	**SE**	**t**	**p**	**β**	**F**	**p**	**R²**	**Adj R²**
SQOL-F Score	Constant	88.00	2.25	39.09	<0.001		14.18	<0.001	0.104	0.097
	FUIP (1 = Yes, 0 = No)	−11.62	3.09	−3.77	<0.001	−0,323				

B: Unstandardized coefficient; β: Standardised coefficient; SE: Standard error; Fear coded as 1 = yes, 0 = no; Adj R²: adjusted R-squared.

Simple linear regression showed that FUIP was significantly associated with SQOL-F scores (p < 0.001). The model explained 10.4% of the variance in SQOL-F (R² = 0.104; Adj R² = 0.097; F = 14.18, p < 0.001).

### Limitations

This study has some limitations. The sample was restricted to married, sexually active women aged 40–50. Therefore, the findings may not be generalizable to all women. Data were collected via a self-administered online questionnaire. This may have limited participation to women with internet access and digital literacy. Psychosocial factors and partner perspectives were not assessed, despite their known influence on sexual well-being. Scales assessing pregnancy-related fear in the literature primarily focus on anxieties during a planned or ongoing pregnancy. However, the fear addressed in this study is the FUIP that arises in the pre-pregnancy period and has psychosocial characteristics. The existing scales are considered insufficient to adequately reflect this construct. Therefore, FUIP was measured using a self-report question rather than a validated multi-item scale. This may have limited the comprehensive assessment of all dimensions of FUIP.

## Conclusions

Significant proportion of perimenopausal women experience negatively affects their sexual quality of life to FUIP is exposed in this study. Sexual well-being was significantly associated with educational level, parity, mode of delivery, and subjective birth experience. Especially, the expressed FUIP women had substantially lower SQOL-F scores. Thus, the importance of a holistic approach in women’s health services that simultaneously addresses sexual health and fertility-related concerns could be highlighted. Nursing-led psychoeducational programs, family planning counseling, and sexual health services should be redesigned to meet the specific needs of women during the perimenopausal transition. Future studies should employ longitudinal designs to better understand the evolving psychological factors and relational dynamics, such as partner support. In the menopausal transition, providing personalized contraceptive counseling is very important and necessary. Moreover, sexual health counseling for perimenopausal women must address not only physiological changes but also emotional and cognitive concerns, including the fear of unintended pregnancy.

## References

[1] Liu X, Zhang X, Wang D, Zhou J, Li Y. Investigation of the quality of life and influencing factors among perimenopausal women. Arch Gynecol Obstet. 2025;312(4):1253–65. doi: 10.1007/s00404-025-08116-1 40699304 PMC12414071

[2] Caruso S, Palermo G, Caruso G, Rapisarda AMC. How Does Contraceptive Use Affect Women’s Sexuality? A Novel Look at Sexual Acceptability. J Clin Med. 2022;11(3):810. doi: 10.3390/jcm11030810 35160261 PMC8836660

[3] Liu X-Y, Peng S-Z, Pei M-Y, Zhang P. The effects of physical activity on depression and quality of life in Chinese perimenopausal women. J Affect Disord. 2023;328:153–62. doi: 10.1016/j.jad.2023.02.061 36801423

[4] Higgins JA, Kramer RD, Wright KQ, Everett B, Turok DK, Sanders JN. Sexual Functioning, satisfaction, and well-being among contraceptive users: a three-month assessment from the HER salt lake contraceptive initiative. J Sex Res. 2022;59(4):435–44. doi: 10.1080/00224499.2021.1873225 33560155 PMC8349922

[5] Bozkurt ÖD, Sevil Ü. Menopause and sexual life. Celal Bayar Univ Inst Health Sci J. 2016;3:4.

[6] Başkıran Y, Tanoğlu FB, Uçkan K, Çeleğen İ, Karaçor T. The impact of maternal age distribution on pregnancy-related complications and neonatal outcomes: A single-center retrospective experience. Adiyaman Univ J Health Sci. 2023;9:215–22. doi: 10.30569/adiyamansaglik.1327740

[7] Grandi G, Di Vinci P, Sgandurra A, Feliciello L, Monari F, Facchinetti F. Contraception During Perimenopause: Practical Guidance. Int J Womens Health. 2022;14:913–29. doi: 10.2147/IJWH.S288070 35866143 PMC9296102

[8] Soltes BA. Contraception in perimenopause. Menopause. 2025;32(5):472–4. doi: 10.1097/GME.0000000000002554 40277951

[9] Nunes LRDC, Coutinho FC, Santos VAD. Fear of childbirth: A review of interventions based on cognitive-behavioral therapy. Psicol Teor Prát. 2022;24:1–22.

[10] Saleh SA, Almadani N, Mahfouz R, Nofal HA, El-Rafey DS, Seleem DA. Exploring the Intersection of Depression, Anxiety, and Sexual Health in Perimenopausal Women. Int J Womens Health. 2024;16:1315–27. doi: 10.2147/IJWH.S464129 39100112 PMC11298183

[11] Hill DA, Taylor CA. Dyspareunia in Women. Am Fam Physician. 2021;103(10):597–604. 33983001

[12] Verrilli L, Esposito-Smith M, Williams M. Sexual health and function in menopause and beyond. In: Challenges in Older Women’s Health: A Guide for Clinicians. Cham, Switzerland: Springer International Publishing; 2021. p. 185–99. Available from: https://link.springer.com/chapter/10.1007/978-3-030-59058-1_12

[13] Symonds T, Boolell M, Quirk F. Development of a questionnaire on sexual quality of life in women. J Sex Marital Ther. 2005;31(5):385–97. doi: 10.1080/00926230591006502 16169822

[14] Tuğut N, Gölbaşı Z. Validity and reliability study of the Turkish version of the Sexual Quality of Life-Female Scale. Cumhuriyet Med J. 2010;32:172–80.

[15] Ceylan Ş. Examination of the relationship between sexual quality of life and happiness level in pregnant women. Turk J Health Sci Res. 2023;6:1–13. doi: 10.51536/tusbad.1384860

[16] Stern J, Bodin M, Grandahl M, Segeblad B, Axén L, Larsson M, et al. Midwives’ adoption of the reproductive life plan in contraceptive counselling: a mixed methods study. Hum Reprod. 2015;30(5):1146–55. doi: 10.1093/humrep/dev048 25771220 PMC4400198

[17] Altunbaş N, Gölbaşı Z. The Effect of Sex Education and Counseling Based on the Ex-PLISSIT Model on Sexual Life in Primigravidas. Clinical and Experimental Health Sciences. 2024;14(2):367–76. doi: 10.33808/clinexphealthsci.1305002

[18] Dashti S, Bahri N, Fathi Najafi T, Amiridelui M, Latifnejad Roudsari R. Influencing factors on women’s attitudes toward menopause: a systematic review. Menopause. 2021;28(10):1192–200. doi: 10.1097/GME.0000000000001833 34520416

[19] Pérez-Herrezuelo I, Aibar-Almazán A, Martínez-Amat A, Fábrega-Cuadros R, Díaz-Mohedo E, Wangensteen R, et al. Female Sexual Function and Its Association with the Severity of Menopause-Related Symptoms. Int J Environ Res Public Health. 2020;17(19):7235. doi: 10.3390/ijerph17197235 33022931 PMC7579461

[20] Nazarpour S, Simbar M, Khorrami M, Jafari Torkamani Z, Saghafi R, Alavi-Majd H. The association between sexual function and body image among postmenopausal women: a cross-sectional study. BMC Womens Health. 2021;21(1):403. doi: 10.1186/s12905-021-01549-1 34876095 PMC8650325

[21] Gupta G, Kumari R, Mishra P, Gupta B. Factors Associated with Quality of Life among Menopausal Women in Lucknow - A Cross-Sectional Study. J Midlife Health. 2023;14(4):257–64. doi: 10.4103/jmh.jmh_152_22 38504732 PMC10946687

[22] Kırat S. Sexual Dysfunction in the Life Cycle of Women: Implications for Psychological Health. Healthcare (Basel). 2025;13(11):1268. doi: 10.3390/healthcare13111268 40508881 PMC12154966

[23] Kaya Şenol D, Gözüyeşil E. Evaluation of the quality of sexual life and sexual function in women with grand multiparity. Health Care Women Int. 2024;45(12):1397–410. doi: 10.1080/07399332.2023.2190982 37010487

[24] Anğın AD, Özkaya E, Çetin M, Gün I, Sakin O, Ertekin LT, et al. Comparison of female sexual function and sexual function of their partners between groups of pregnant and non-pregnant women. Ginekol Pol. 2020;91(5):235–9. doi: 10.5603/GP.2020.0062 32495927

[25] Baud D, Sichitiu J, Lombardi V, De Rham M, Meyer S, Vial Y. Comparison of pelvic floor dysfunction 6 years after uncomplicated vaginal versus elective cesarean deliveries: A cross-sectional study. Sci Rep. 2020;10:1–8. doi: 10.1038/s41598-020-78625-3 33299112 PMC7726103

[26] Terece C, Turan G, Uçkan HH, Eser A, Özler MR, Dunyamalıyeva A, et al. Impact of delivery mode on sexual functions in the first year after childbirth: A comparative study of vaginal and cesarean sections. Thai J Obstet Gynaecol. 2024;:365–76. doi: 10.14456/tjog.2024.40

[27] Fanshawe A-M, De Jonge A, Ginter N, Takács L, Dahlen HG, Swertz MA, et al. The Impact of Mode of Birth, and Episiotomy, on Postpartum Sexual Function in the Medium- and Longer-Term: An Integrative Systematic Review. Int J Environ Res Public Health. 2023;20(7):5252. doi: 10.3390/ijerph20075252 37047868 PMC10094321

[28] Calpbinici P. The relationship between traumatic childbirth perception, desire to avoid pregnancy, and sexual quality of life in women. Int J Gynaecol Obstet. 2024;167(1):265–72. doi: 10.1002/ijgo.15797 39016294

[29] Geleta D, Kebede A, Bulcha G, Usman H, Hajibediru K, Kebede S, et al. Prevalence and Predictors of Contraceptive Use Among Women of Premenopausal Period in Ethiopia: A Retrospective Cross-Sectional Data Analysis. Open Access J Contracept. 2021;12:149–56. doi: 10.2147/OAJC.S318486 34349572 PMC8326935

[30] Phan TC, Hoang LB, Tran TK, Pham TTT, Bui AV, Dao HT, et al. Fear-Related Reasons for Avoiding Sexual Intercourse in Early Pregnancy: A Cross-Sectional Study. Sex Med. 2021;9(6):100430. doi: 10.1016/j.esxm.2021.100430 34628113 PMC8766271

[31] Saha S, Lim CC, Cannon DL, Burton L, Bremner M, Cosgrove P, et al. Co-morbidity between mood and anxiety disorders: A systematic review and meta-analysis. Depress Anxiety. 2021;38:286–306. doi: 10.1002/da.23113 33225514 PMC7984258

[32] Rahmani A, Afsharnia E, Fedotova J, Shahbazi S, Fallahi A, Allahqoli L, et al. Sexual Function and Mood Disorders Among Menopausal Women: A Systematic Scoping Review. J Sex Med. 2022;19(7):1098–115. doi: 10.1016/j.jsxm.2022.03.614 35752457

[33] Armeni A, Armeni E, Augoulea A, Stergiotis S, Kaparos G, Alexandrou A, et al. Climacteric symptoms, age, and sense of coherence are associated with sexual function scores in women after menopause. J Sex Med. 2023;20(3):313–23. doi: 10.1093/jsxmed/qdac031 36763958

[34] Burgin J, Bailey JV. Factors affecting contraceptive choice in women over 40: a qualitative study. BMJ Open. 2022;12(11):e064987. doi: 10.1136/bmjopen-2022-064987 36414297 PMC9685181

[35] Lega IC, Jacobson M. Perimenopause. CMAJ. 2024;196:E1169. doi: 10.1503/cmaj.240337 PMC11482657 39406411

