# Supplementary material for: Fear of unintended pregnancy and sexual quality of life during the menopausal transition in a Turkish population: A cross-sectional study of associated factors
Source: PLoS One. 2026 Jul 14;21(7):e0353656. doi: 10.1371/journal.pone.0353656 (PMC13367739; doi:10.1371/journal.pone.0353656)
Supplement: Attachment — Submitted filename: Fear of unintended pregnancy and sexual quality of life.docx [file pone.0353656.s001.docx]

Thank you for asking me to review this interesting manuscript titled “ Fear of Unintended pregnancy and sexual quality of life during the menopausal transition in a Turkish population: A cross-sectional study of associated factors”. This topic addresses an important issue concerning perimenopausal women and their sexual health in Turkey.

This manuscript is well-conceived and was administered using the Google form, making the study unique. However, as it stands now, the manuscript needs to be improved upon for one to appreciate the study.

The authors need to pay attention to the language, as this is of concern. A lot of times, one cannot appreciate the message, and a couple of times, so much is said without communicating. This has prevented a thorough evaluation of the manuscript. Nevertheless, I will make some specific comments:

1. Under the introduction – line 37, the definition of perimenopause should be referenced. Interestingly, another definition is written in lines 62–63 of perimenopause, without reference to the previous statement. Kindly reconcile this.
2. The second paragraph, especially lines 45 -50 are not clear
3. The third paragraph is not clear and is not connected to the previous paragraph.
4. The justification should be concise, and the objectives well outlined
5. Under materials and method-

- Sample size determination is unclear. How did you arrive at 122? Did you consider attrition?
- What is G*Power?
- Participant recruitment is not clear
- Nothing was mentioned about the study setting, study population
- Study
- Line 125 should be Research Instrument. Reconcile it with line 135 and make it explicit
- Lines 165 & 166 are part of the result
