# Supplementary material for: Fear of unintended pregnancy and sexual quality of life during the menopausal transition in a Turkish population: A cross-sectional study of associated factors
Source: PLoS One. 2026 Jul 14;21(7):e0353656. doi: 10.1371/journal.pone.0353656 (PMC13367739; doi:10.1371/journal.pone.0353656)
Supplement: Attachment — Submitted filename: Response to Reviewers.docx [file pone.0353656.s002.docx]

RESPONSE TO REVIEWERS

1. Three separate files have been prepared and uploaded: (1) the “*Response to Reviewers”*, (2) the “*Revised Manuscript with Track Changes”*, and (3) a clean version of the revised manuscript without tracked changes, which has been labeled as “*Manuscript.”*

2. The ethics statement has been removed from all sections other than the Methods section and is now presented only in the Methods section (Page 19, Lines 386–389), in accordance with the journal’s guidelines.

3.The manuscript has been carefully revised to comply with PLOS ONE’s style requirements, including formatting and file naming, in accordance with the provided templates (Lines 3, 5, 8, 9, and 11–14 have been deleted).

4.The statement “Competing Interests: The authors declare that they have no conflict of interest. The data supporting the findings of this study are available from the author upon reasonable request.” has been added to the cover letter and incorporated into the manuscript.

5. In accordance with PLOS ONE’s Data Policy, a non-author institutional point of contact has been provided to ensure long-term data accessibility. Data requests can be directed to the Kahramanmaraş Sütçü İmam University Faculty of Medicine Clinical Research Ethics Committee via the following contact details: ksukaek@ksu.edu.tr, Tel: +90 344 300 34 24. In addition, the Data Availability Statement has been revised accordingly to comply with the journal’s requirements.

6. Response to Reviewer 1: Thank you for your valuable comment. A reference has been added to the definition of perimenopause in line 37. Additionally, the statement in lines 62–63 has been revised to function as a transitional sentence rather than a separate definition, ensuring consistency throughout the text.

7. Thank you for your valuable comment. The second paragraph (lines 45–50) has been revised to improve clarity, readability, and overall language. The sentences have been simplified and reorganized to ensure a clearer and more coherent presentation of the content.

8. I thank the reviewer for this valuable comment. In response, the third paragraph has been carefully revised to improve clarity and coherence. Specifically, it has been restructured to establish a clearer conceptual link with the preceding paragraph by explicitly framing unintended pregnancy risk as a component of sexual health during the perimenopausal period. Repetitive statements have been removed, and the flow of ideas has been improved to ensure a more logical progression from sexual health and quality of life to pregnancy-related risks and their physical and psychological consequences.

9. I thank the reviewer for these valuable comments. All suggested revisions have been addressed under the Materials and Methods section. Specifically, the study design, sample size calculation, participant recruitment process, study setting and population, and research instruments have been clarified and reorganized for better transparency and consistency.

10. The Introduction section has been revised by reducing redundancy and removing unnecessary sentences. Repetitive expressions have been eliminated, and the text has been streamlined to improve clarity and overall flow.

11. I thank the reviewer for this helpful comment. The Results section has been revised by shortening the descriptions of the tables and removing repetitive details. The text now focuses on summarizing the most relevant and statistically significant findings.
