# Supplementary material for: Fear of unintended pregnancy and sexual quality of life during the menopausal transition in a Turkish population: A cross-sectional study of associated factors
Source: PLoS One. 2026 Jul 14;21(7):e0353656. doi: 10.1371/journal.pone.0353656 (PMC13367739; doi:10.1371/journal.pone.0353656)
Supplement: Attachment — Submitted filename: Response_to_Reviewers_auresp_2.docx [file pone.0353656.s003.docx]

Dear Editor-in-Chief and Reviewers,

I would like to thank you for your insightful comments and valuable suggestions. I have carefully considered all reviewer comments and made all possible revisions accordingly. The changes made to the manuscript are detailed in the table below.

I sincerely appreciate the time and effort you invested in reviewing my manuscript. Your constructive feedback has significantly contributed to improving the quality and clarity of the paper. Thank you for this suggestion. The manuscript has been thoroughly revised to improve English language quality and readability. The specific revisions are detailed below.

Thank you again for your valuable time, expertise, and thoughtful recommendations.

Yours sincerely,

Aysun Badem

| **Reviewer** | **Comment** | **Response to comment** | **Page Number** |
| --- | --- | --- | --- |
| **Reviewer 1** | 1. Lines 9&10 are unclear, kindly rewrite them | - Thank you for this comment. The objective statement has been revised for clarity and grammatical accuracy. | Page 1 |
|  | 2. Lines 11 &12 - incomplete and should be rewritten | - Thank you for this comment. The methods section of the abstract has been rewritten to improve clarity and readability. | Page 1 |
|  | 3. Lines 15 -18 - unclear | Thank you for this observation. The wording of the results section has been refined to improve clarity and ensure accurate interpretation of the study findings. | Page 1 |
|  | 4. Lines 32 - 35 - not clear | Thank you for this observation. The sentence has been revised and simplified for greater clarity. | Page 2 |
|  | 5. lines 51 - 52 should be referenced. | Thank you for this suggestion. Appropriate references have been added to support the statements in the indicated section. | Page 3 |
|  | 6. line 67 ---some woman may experience -- incorrect | Thank you for identifying this error. The incorrect word usage has been corrected in the revised manuscript. | Page 3 |
|  | 7. line 77 - 78 - not clear | Thank you for this observation. The sentence has been rephrased to present the intended meaning more clearly. | Page 4 |
|  | 8. line 81 - a word is missing in the statement | Thank you for identifying this omission. The missing word has been added to the sentence. | Page 4 |
|  | 9. line 92- 94 - recruitment of participants not clear | Thank you for this comment. Additional information has been provided to clarify the participant recruitment process and data collection procedure. | Page 4 |
|  | 10. line 99 - define " sexually active" | Thank you for this comment. To avoid ambiguity and provide a clearer description of participant eligibility, the term “sexually active” was clarified by adding the definition “currently engaging in sexual intercourse” to the inclusion criteria. | Page 5 |
|  | 11. line 102 - statement is unclear | Thank you for this observation. The sentence has been revised to clarify the participant recruitment and inclusion process. | Page 5 |
|  | 12. line 105 - define physical disability | Thank you for this comment. The term “physical disability” was clarified as physical disabilities that could affect sexual functioning. | Page 5 |
|  | 13. line 110 - rewrite for clarity | Thank you for this comment. To improve clarity and readability, the sentence was divided into two parts and the study variables were presented in a more structured manner. | Page 5 |
|  | 14. line 114/1115- " mode of contraceptive method use" - do you mean type | Thank you for this observation. The term “mode of contraceptive method use” has been revised to “type of contraceptive method used” for greater precision and clarity. | Page 5 |
|  | 15. line 144/145 - paraphrase this statement | Thank you for this comment. The statement has been rephrased to improve its academic style and clarity. | Page 6 |
|  | 16. Line 327 - 330 is not clear. | Thank you for this comment. The statement regarding the generalizability of the findings has been revised to improve clarity and accurately reflect the characteristics of the study sample. | Page 17 |
|  | 17. Under limitation, kindly explain why data collection through an online platform is a limitation. | Thank you for this valuable comment. An additional explanation has been added to clarify that online data collection may have limited participation to women with internet access and adequate digital literacy. | Page 17 |
| **Reviewer 2** | Thank you for addressing the comments for your manuscript. I see all of my recommendations were addressed carefully. | Thank you for your positive feedback. I sincerely appreciate your careful review and valuable comments, which helped improve the quality and clarity of the manuscript. |  |
